# Good Liars Are Neither ‘Dark’ Nor Self-Deceptive

**DOI:** 10.1371/journal.pone.0127315

**Published:** 2015-06-17

**Authors:** Gordon R. T. Wright, Christopher J. Berry, Caroline Catmur, Geoffrey Bird

**Affiliations:** 1 Department of Psychological Sciences, Birkbeck, University of London, London, WC1E 7HX, United Kingdom; 2 School of Psychology, Plymouth University, Drake Circus, Plymouth, PL4 8AA, United Kingdom; 3 Department of Psychology, University of Surrey, Guildford, GU2 7XH, United Kingdom; 4 Institute of Cognitive Neuroscience, Queens Square, London, WC1N 3AR, United Kingdom; 5 MRC Social, Genetic & Developmental Psychiatry Centre, Institute of Psychiatry, London, SE5 8AF, United Kingdom; University of Bologna, ITALY

## Abstract

Deception is a central component of the personality 'Dark Triad' (Machiavellianism, Psychopathy and Narcissism). However, whether individuals exhibiting high scores on Dark Triad measures have a heightened deceptive ability has received little experimental attention. The present study tested whether the ability to lie effectively, and to detect lies told by others, was related to Dark Triad, Lie Acceptability, or Self-Deceptive measures of personality using an interactive group-based deception task. At a group level, lie detection accuracy was correlated with the ability to deceive others—replicating previous work. No evidence was found to suggest that Dark Triad traits confer any advantage either to deceive others, or to detect deception in others. Participants who considered lying to be more acceptable were more skilled at lying, while self-deceptive individuals were generally less credible and less confident when lying. Results are interpreted within a framework in which repeated practice results in enhanced deceptive ability.

## Introduction

Many studies have shown humans to be relatively poor lie detectors, performing little better than chance at discriminating truthful from deceptive statements [1,2]. Despite the poor performance of the average individual, some authors claim substantial individual differences, such that some people are capable of detecting deception at levels far above chance (e.g. the ‘Wizards Project’ of O'Sullivan & Ekman, [3]; c.f. Bond & DePaulo, [2]). This claim has prompted a series of studies aiming to elucidate the factors which may determine deception detection ability. The results of these studies are fairly conclusive; of the characteristics studied (age, occupation, education or gender), none seem to consistently co-vary with the ability to detect lies (see meta-analyses [4,2]).

Recent research from our own lab suggests that lie detection ability may be associated with lie production ability—a deception-general ability. Using an interactive paradigm (the **Dece**ptive **I**nteraction **T**ask ‘DeceIT’ [5]), we found that participants able to successfully deceive others were also able to successfully detect others’ attempts to deceive. However, this result requires replication, as it stands in contrast to two earlier studies which, using non-interactive tasks, found no relationship between lie production and detection [6,7].

Importantly, relatively few studies have examined predictors of the ability to *produce* successful lies. This is surprising as the success or failure of any deceptive interaction may be more attributable to the performance of the liar than that of the lie detector [8]. A meta-analysis by Bond and DePaulo [2] suggested that individuals vary more in terms of the detectability of their lies rather than in their ability to detect deception, and vary maximally in terms of their general credibility (or Demeanour Bias; [9]).

Despite limited experimental attention, it is often assumed that there exists an association between the ‘Dark Triad’ and deceptive ability, with those scoring higher on Dark Triad measures demonstrating increased ability to deceive others. The Dark Triad is a cluster of three higher-order personality constructs (Machiavellianism, Psychopathy and Narcissism), which are moderately inter-correlated, but which are nevertheless considered to be distinct [10]. The Dark Triad is stable over time, observed across various regions of the world [11], and is moderately heritable (with heritability estimates between .31 and .72; [12]).

The Dark Triad has been associated with numerous antisocial tendencies related to deceit (e.g. [13,14,15]). Machiavellians prioritise the attainment of money and power [16], and have been described as preferentially adopting deceitful and duplicitous behaviour in order to gain dominance [17]. This preference for deceptive strategies is supported by diary studies in which Machiavellians report telling more lies than those low in Machiavellianism [18]. However, experimental studies of the relationship between deceptive ability and Machiavellianism have produced mixed results. An early study by Exline, Thibaut, Hickey, and Gumpert [19] found that high Machiavellians were able to lie more convincingly than low Machiavellians. Five further studies, however, failed to show the same pattern of results [20]. DePaulo and Rosenthal [6] used a video-based deception task and found that high Machiavellian individuals were less likely to be caught lying than low Machiavellian individuals, but this result was not replicated by Manstead, Wagner and MacDonald [7]. In the only study also to look at lie detection ability, Geis and Moon [21] found that high Machiavellians were better able to lie than low Machiavellians, and were rated as more credible, but did not show enhanced lie detection abilities. A meta-analysis of this mixed literature found no evidence for enhanced deceptive skill in high-Machiavellian individuals [22].

The second Dark Triad trait, psychopathy, has been argued to be the prototypical syndrome for pathological lying, deception, and manipulation [23,24,25,26], with psychopaths deriving particular satisfaction from deceit [25,27]. There have not been many empirical studies of the association between deception production abilities and psychopathy, but the existing literature suggests that although psychopaths report a higher ability to deceive others than non-psychopaths, this may not be an accurate reflection of reality [28,29]. In addition, no relationship was found between psychopathy and the ability to detect deception [25,30] although in the latter study the correlation between deception detection and psychopathic traits approached significance.

Narcissism is not associated with deception as commonly as the rest of the Dark Triad traits. Narcissists are competitive, seek power and glory, and exhibit a grandiose sense of self. Their grandiose sense of self is maintained in the face of negative/realistic feedback through self-deception [31,32,10,33,34]. The ability to successfully self-deceive has been argued to bring about a greater ability to deceive others (see Trivers, [35,36]). Indeed, Trivers [36] has argued that the reason why humans have evolved the capacity to self-deceive is precisely because it aids in the deception of others; if the liar is unaware that they are lying it is less likely that they will exhibit any deceptive ‘cues’. However, whether narcissists do indeed have greater deceptive ability than non-narcissists has not been investigated empirically.

This study aims to examine the relationship between deceptive ability (both production and detection) and the Dark Triad, including associated measures of lie acceptability and self-deception. The overall aim is to determine whether any of these measures can predict performance when producing or detecting deceptive statements. The use of the DeceIT procedure allows these individual difference variables to be tested against success in the detection and the production of deceptive statements, and also against measures of general credibility and credulity (or truth bias) through the use of signal detection theory [37] applied to performance in both the Sender and Receiver roles as originally presented in [5] and detailed in [38].

## Method

### Participants

75 healthy adult participants (28 male, 47 female, Mean age = 27.25 years, SD = 7.59) took part in a computer-administered competitive interactive group deception task (DeceIT—Wright et al., 2012 [5]). Participants were fluent English speakers and all provided written informed consent to participate. The procedure received ethical approval from the Birkbeck Psychological Research Ethics Committee.

### Materials and apparatus

Prior to the task, participants completed questionnaires assessing Machiavellianism, sub-clinical Narcissism and sub-clinical Psychopathy. These were: the MACH-IV [20], the Narcissistic Personality Inventory Short-Form (NPI-16) [39] and the Sub-Clinical Self-Report Psychopathy Questionnaire Short-Form (SRP-SF) [40]. Participants also completed the Self-Deception Scale of the Balanced Inventory of Desirable Responding [41] as used by Lynch & Trivers [42] to measure self-deception, and the Lie Acceptability Scale [43] which measures the extent to which an individual considers deceit to be an acceptable strategy to achieve personal goals.

As per the original DeceIT task detailed in [5] a False-Opinion Paradigm was employed [44,45]. Ground truth was initially obtained by presenting a “Social Opinion Questionnaire” which comprised a series of ‘For or Against’ questions relating to topical issues that had recently featured in mainstream media such as Censorship of the Media and Nuclear Power.

In a development from its earlier implementation, the DeceIT paradigm was administered on 9-inch tablet PCs. The underlying procedure (see below) was otherwise identical to the original DeceIT task [5]. Experimental instructions and stimulus material were presented on the tablet PCs, which were positioned for ease of viewing and were not visible to other players. Participant responses in each trial were prompted on screen and collected via touchscreen responses.

### Procedure

Participants were recruited in groups of five to a “Communication Skills Experiment”. Participants were seated in a circle of five chairs with integral writing platforms. The participants were identified by numbers from one to five and informed that they would be referred to by number only to maintain confidentiality.

The deceptive interactive task (DeceIT) required participants to take turns making true or false statements on the opinion topics previously surveyed using the Social Opinion Questionnaire. Upon a cue presented on the tablet screen, the participant randomly allocated to the Sender role for the current trial was informed of the topic (e.g. Animal Testing) and the veracity of the statement required (lie or truth). All those allocated to the Receiver role (i.e. those required to judge the veracity of the statement) were told to attend to a specified participant by number. The statements were made verbally and participants were instructed to speak as soon as they were ready for between 20 and 30 seconds. At the end of the statement, Senders were asked to rate the perceived credibility of the statement they had just made. The four participants not randomly allocated to the Sender role on each trial (i.e. those in the Receiver role) were instructed to judge whether the Sender was making a true or false statement.

The experimental trials continued with quasi-random allocation of topics and of the Sender role until each participant had made both truthful and deceptive statements on each of the 8 opinion topics, resulting in 80 live trials per participant group. High-value incentives (£50 monetary prizes) were offered to the best performers in the Sender and Receiver roles. In order to accurately measure credibility, it was made clear to participants that to be as successful as possible in the Sender role they should be as credible as possible on every trial—and that trying to appear as if they were lying when telling the truth would be a counter-productive strategy.

Prior to the DeceIT task all participants observed the experimenter perform two demonstration trials to the group as a whole (reading out verbatim responses from previous iterations of the task), and ran through two practice trials (one of which required them to lie, and one to tell the truth) without speaking aloud. At the end of the task, participants rated their Guilt, Anxiety and Cognitive Load (described as mental effort in the rubric) in both experimental conditions on 7 point Likert scales with end points labelled ‘not at all’ to ‘extremely’.

### Data collection and analysis strategy

Performance in the Receiver and Sender roles was analysed using a Signal Detection Theory framework (SDT) [37] as previously described in Wright et al. [5]. Performance in both Receiver and Sender roles is indexed using lie-truth differential performance (*d*ʹ) measured independently of bias (C). Separate SDT measures were calculated for the Receiver/Sender roles: the Receiver’s capacity to discriminate lies from truths is indexed by *d*ʹ_Receiver_; the corresponding measure of bias, *C*_Receiver_, corresponds to Truth Bias (with negative values indicating the tendency to judge statements as truthful regardless of veracity, while a positive value indicates a bias to classify messages as lies). The discriminability of the Sender’s truths and lies is indexed by *d*ʹ_Sender_. The corresponding measure of bias, *C*_Sender_, indicates the perceived overall credibility of a Sender, regardless of their veracity, and is often termed ‘Demeanour Bias’ within the deception literature—negative scores indicating higher credibility and positive scores indicating lower credibility. With these measures, better lie detection is indicated by higher *dʹ*_Receiver_ values, and increasingly successful deception (relative to success when telling the truth) is indicated by more negative values of *dʹ*_Sender_.

## Results

### Dark Triad measures

Broad variability in all Dark Triad measures was observed, with 31 individuals (41% of sample) being identified as “high-Machiavellians”. Recommended cut-off scores for Narcissism and Psychopathy are not available given the sub-clinical usage of the instruments employed. In line with previous research [10] moderate correlations were found between all Dark Triad components ranging from .247 to .349 (all p<.05, see Table 1 below). In addition, a significant positive correlation was observed between Machiavellianism and Lie-Acceptability (.384, p = .001) and, between Narcissism and Self-Deception (.259, p = .025).

**Table 1:** Table of correlations between Machiavellianism, Narcissism, Psychopathy, Composite Dark Triad, Lie Acceptability, Self-Deception and the four SDT-derived performance measures in the DeceIT task.

	Machiavellianism	Narcissism	Psychopathy	Lie Acceptability	Self-Deception
Machiavellianism	x	.260, p = .024*	.349, p = .002*	.384, p = .001*	.118, p = .314
Narcissism		x	.247, p = .033*	-.013, p = .909	.259, p = .025*
Psychopathy			x	.101, p = .386	.157, p = .179
Lie Acceptability				x	-.093, p = .429
Self-Deception					x
*d’*_Receiver_	-.026, p = .828	-.145, p = .216	-.059, p = .617	-.022, p = .854	-.196, p = .092
*C*_Receiver_	.094, p = .422	.113, p = .333	.082, p = .484	.038, p = .747	-.043, p = .713
*d’*_Sender_	-.103, p = .379	.054, p = .646	-.063, p = .593	-.245, p = .034*	.131, p = .261
*C*_Sender_	-.098, p = .404	.179, p = .123	.045, p = .702	-.093, p = .426	.256, p = .027*

(* indicates significant at p < 0.05).

### DeceIT measures

**Fig 1:**
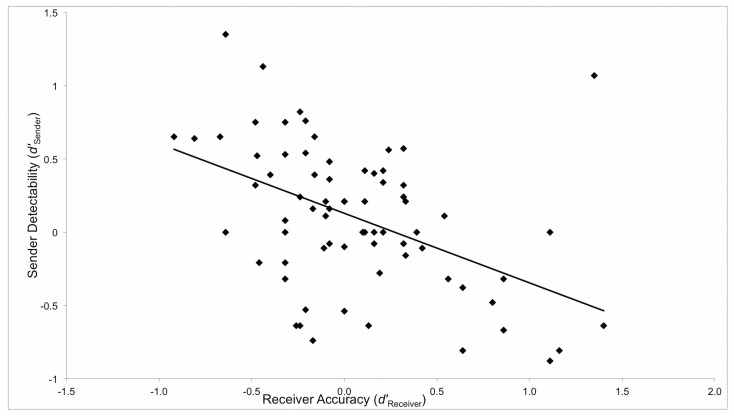
Correlation between Sender and Receiver performance using SDT measures for Receiver Accuracy (*dʹ Receiver*) and Sender Detectability (*dʹ Sender*): *r* = − 0.471, *p* <.001.

Broad individual differences were observed in all four of the performance measures (M *dʹ*_Receiver_ = 0.078, SD = 0.496; M *C*_Receiver_ = 0.065, SD = 0.193; M *dʹ*_Sender_ = 0.091, SD = 0.502; M *C*_sender_ = -0.086, SD = 0.191). As previously observed [5] detectability in the Sender role (*dʹ*_Sender_) and the ability to discriminate in the Receiver role (*dʹ*_Receiver_) were significantly correlated (*r* = −0.471, p <.001, Fig 1). As the ability to discriminate truthful from deceptive messages increased, the ability to produce deceptive messages that were less likely to be judged as deceptive in comparison to truthful messages, increased. This replicates the main finding presented in [5].

Participants reported greater Guilt, Anxiety and Cognitive Load when lying than when telling truth (Guilt *t*_*(74)*_ = 8.029, p <.001, Anxiety *t*_*(74)*_ = 7.257, p <.001, Cognitive Load *t*_*(74)*_ = 7.588, p <.001), and exhibited typical chronometric cues to deception (e.g. [46,47,5]). Response latency was significantly shorter when participants told the truth (M = 4.128 s SD = 1.336) than when they lied (M = 5.510 s SD = 2.371, *t*_*(74)*_ = -6.409, p <.001), while response duration was significantly longer for truthful statements (M = 21.778 s, SD = 6.178) than for deceptive statements (M = 16.736 s, SD = 3.150, *t*_*(74)*_ = 6.818, p<.001). Decreasing detectability in the Sender role (*dʹ*_Sender_) was associated with a reduced response latency difference between truthful and deceptive statements (r = 0.237, p = .040), as observed in Wright et al. [5].

To permit comparison with previous studies, performance was also analysed using percentage accuracy. Overall accuracy in the Receiver role was found to be 51.49% (SD = 9.39), not significantly different from chance (*t*_*(74)*_ = 1.373, p = .174). To compare truth-bias in the Receiver role with previously reported findings we calculated the number of statements of all types classified by Receivers as truthful and found it to be 52.47% (SD = 7.35%) a figure significantly higher than chance (*t*_*(74)*_ = 2.912, p = .005).

### Associations between individual differences and DeceIT performance measures

No association was observed between any of the Dark Triad measures (Machiavellianism, Narcissism or Psychopathy; see Table 1), or a combined Dark Triad score (r = .133, p = .254), and performance in either the Sender or Receiver roles in the DeceIT task. However, a significant correlation was observed between Lie Acceptability Scale scores and discriminability in the Sender role (*dʹ*_Sender_, r = -.245, p = .034); those who consider deception more acceptable tended to make deceptive statements that were *more* difficult to discriminate from the truth by Receivers, while a significant correlation was found between Self-Deception and credibility in the Sender role (*C*_Sender_, r = .256, p = .027) indicating that individuals higher in the trait of self-deception appeared generally *less* credible in the task, i.e. overall their lies and truths were less likely to be believed. Step-wise multiple regressions using Dark Triad scores as predictor variables, and Sender and Receiver performance in the DeceIT task as dependant variables were conducted, but failed to reveal any higher-order relationships between Dark Triad scores and deceptive ability.

Participants’ self-reported confidence ratings were significantly lower for the deceptive condition (M = 0.468, SD = 0.180) than for the truthful condition (M = 0.603, SD = 0.207, t(74) = -4.762, p <.001). The difference between confidence ratings for deceptive and truthful conditions (where lower scores indicate lower relative confidence in the lie condition) was found to correlate negatively with *C*_Sender_ (r = -.253, p = .029) and positively with self-deception (r = -.312, p = .006). Given that *C*_Sender_ (overall credibility) is derived such that lower scores indicate *higher* credibility, the negative relationship with confidence indicates that participants with higher overall credibility in the DeceIT task were more confident. The positive relationship between self-deception and relative confidence when lying indicates that increasing self-deception is associated with less confidence when lying in comparison to when telling the truth. This relationship was driven by confidence when lying: individuals high in self-deception (by median split) were significantly less confident in the deceptive condition (M = 0.416, SD = 0.174) than those low in self-deception (M = 0.520, SD = 0.173, t(73) = -2.595, p = .011), whereas no significant difference was observed for their confidence when telling the truth.

## Discussion

The main finding of this study is that Dark Triad traits (Machiavellianism, Psychopathy or Narcissism) were not associated with the ability to either produce lies which others found difficult to discriminate from truth, or to discriminate truth from lies when judging others. However, Lie Acceptability was associated with the ability to produce successful lies. In addition, the extent to which one engages in self-deception was found to correlate with poorer overall performance in the Sender role of the DeceIT task as measured by credibility (*C*_Sender_).

From the characterisation of the ‘Dark Triad’ personality traits we expected to find that individuals scoring highly on such personality traits would show a greater deceptive ability. In contrast however, none of the individual Dark Triad measures were associated with either the ability to produce or to detect deception. These results are consistent with the extant research on Dark Triad traits and deceptive ability which have predominantly used non-interactive paradigms: although some previous research has claimed a link between Machiavellianism and deceptive ability (e.g. [19,6]), a meta-analysis of existing research found no support for an increased ability to lie in high Machiavellian individuals [22]; similarly the little empirical research conducted on the ability of psychopathic individuals to deceive has identified no particular deception-related ability [25,30]. As far as we are aware no previous study has looked at the deceptive ability of individuals with narcissistic traits.

A potential explanation for the inconsistent findings with regard to an association between Machiavellianism and the ability to produce successful lies may be found in the significant correlation between the degree to which participants rated lying as acceptable and their ability to produce successful lies. Machiavellianism and Lie Acceptability were significantly correlated (an acceptance of deception as a means of achieving one’s goals is one of the primary features of the Machiavellian trait). This correlation was only of moderate strength however (r = .384) and so sampling bias in terms of Lie Acceptability within a high Machiavellian sample may determine the extent to which the high Machiavellian sample outperform a low Machiavellian sample via the mediating influence of Lie Acceptability. More generally, the association between Lie Acceptability and success in producing lies merits further investigation. At least two mechanisms may explain why those for whom lying is more acceptable are better able to lie. First, the endorsement of lying as an interpersonal strategy may permit an individual to lie more, and thus garner greater opportunities to practice deceiving others. Second, those who consider lying more acceptable might experience less of the guilt and anxiety brought about by lying [48] and therefore exhibit fewer associated cues. The second hypothesis received little support from the current data however, where the relationships between lie acceptability measures and self-reported guilt and anxiety when lying were not significant (analysed using differential scores, lie minus truth, or lie alone, max r = 0.111, p = .342).

An experiential account is also likely to explain the association between *C*_Sender_ and the confidence with which people lie (relative to their confidence when telling the truth). *C*_Sender_ indexes an individual’s general credibility (or Demeanour Bias)—irrespective of whether they are telling the truth or lying. Individuals with higher credibility reported higher relative confidence when lying than those with lower credibility. Sender demeanour has been shown to have a strong influence on the outcome of deceptive encounters [49], and the relationship between credibility and confidence may be an indication that individuals have learned their level of credibility over many deceptive encounters.

Self-deception was not associated with an increased ability to lie effectively in these data: no relationship was observed between the ability to deceive as indexed by *dʹ*_Sender_—and the relationship between self-deception and general credibility was significantly *negative*. Thus, individuals high in self-deception were seen as generally less credible than those low in self-deception. This finding is in contrast to the hypothesis that self-deception contributes to deceptive success (e.g. [36,50], and with a recent investigation in a classroom setting [51]; however note that in this study participants were not actually lying, others were deceived by the participant’s erroneous, but not deceptive, self-perception). Self-deception was hypothesized to be of benefit when lying in two ways. First, the self-deceptive individual no longer emits consciously-mediated cues to deception (such as signs of nervousness, guilt, or cognitive load) as they are not aware that they are lying. Second, those who are self-deceptive are able to project an image of themselves as being more confident than they may otherwise seem (due to the ability to deceive themselves about their strengths and weaknesses), and gain the resultant social advantages (including being viewed as credible) that self-deception brings. The current data does not provide any support to the first hypothesis; the ability to deceive as measured by *dʹ*_Sender_ was not associated with self-deception, and the negative relationship between *C*_Sender_ and self-deception goes against the second hypothesis, as individuals higher in self-deception were generally perceived as being less credible. Interestingly, this effect may be mediated by confidence. In contrast to the hypothesized relationship between self-deception and confidence when lying, those high in self-deception had lower relative confidence that their lies would be believed (r = -.312, p = 0.006).

Of note is the replication of the association between skill in producing successful lies and in detecting the lies of others. This result was first found using the DeceIT paradigm [5], but required replication as two previous studies [6,7] had found lie production and detection skill to be unrelated (although it should be noted that the correlation between lie production and detection approached significance in the latter study). A possible reason for the discrepancy between the results obtained using the DeceIT procedure and the earlier studies is that the DeceIT procedure involved social interaction whereas the earlier studies did not. It is therefore plausible that whatever skill contributes to the correlation between detection and production of deception in the DeceIT paradigm is of maximum efficacy when social interaction is possible.

In both studies using the DeceIT paradigm a significant correlation was observed between discriminability when lying and an individual’s increase in response time when lying. These data therefore suggest that when detecting deception, Receivers (lie detectors) use response latency as a cue to deception, and that good liars can control the extent to which they exhibit this cue. The use of response latency as a cue to deceit by those judging deception is valid; response latencies for deceptive statements were significantly longer than those for true statements. Whether the use / control of response latency by Receivers / Senders is deliberate or implicit is an interesting avenue for future investigation.

As in any experimental study of deception one must question the validity of the deceptive behaviour elicited by the experimental context. It could be argued that in the DeceIT paradigm the experimenter sanctions lies, and therefore the guilt associated with deception is reduced. We have discussed this issue, and the wider issue of laboratory-based deception research previously [5], and interested readers are directed to this paper. We briefly note that previous work suggests that there is very little difference in the detectability of sanctioned and unsanctioned lies (see meta-analysis by Sporer & Schwandt, [52]), and that participants were attempting to deceive other participants in a competitive scenario, rather than attempting to deceive the experimenter who had sanctioned the lie. While stakes were not of the magnitude involved in real-life criminal investigations, the availability of significant financial prizes and the competitive element of the task were designed to increase the likelihood that participants were engaged in the task.

In summary, despite the way in which its component traits are conceptualised, the Dark Triad appears unrelated to deceptive ability, either in the role of lie detector or liar. Although the current experimental sample (n = 75) is modest in comparison with individual differences questionnaire based research samples, sufficient variability was observed in Dark Triad traits to suggest any meaningful relationship with deceptive performance might have emerged. Indeed, power analysis suggests even the strongest relationship between Dark Triad measures and deceptive ability would require a sample of 191 participants in order to reach significance and would suggest poorer deception detection associated with Dark Triad traits rather than any deceptive skill.

In contrast, lie acceptability, the extent to which one endorses deceit and manipulative behaviour, relates positively to deceptive success. Furthermore, contrary to the hypothesis that self-deception is evolutionarily selected to promote the effective deception of others, these results suggest that high self-deceivers are less credible overall, and less confident when lying, than those low in self-deception. This study replicated the key findings of Wright et al. [5]: the ability to lie well correlates with an ability better to detect deception in others; and the control of response latency difference when lying may be key to producing successful lies, and to detecting those lies in others.
